# Case Report: Spinoglenoid notch cysts associated with type II SLAP lesions: a report of two cases and review of the literature

**DOI:** 10.3389/fsurg.2026.1792792

**Published:** 2026-10-01

**Authors:** Jianan Ouyang, Weiming Guo, Fen Luo

**Affiliations:** 1 Department of Sports Medicine, Affiliated Nanshan Hospital of Shenzhen University, Shenzhen, China; 2 Department of Orthopaedics, Affiliated Hospital of Guilin Medical University, Guilin, China; 3 Department of Orthopaedics, The Second Affiliated Hospital of University of South China, Hengyang, China

**Keywords:** arthroscopic surgery, case report, indirect decompression, labral repair, SLAP lesion, spinoglenoid notch cyst, suprascapular neuropathy

## Abstract

**Background:**

Spinoglenoid notch cysts associated with type II superior labrum anterior-to-posterior (SLAP) lesions are an uncommon cause of suprascapular nerve compression.

**Case Presentation:**

This report presents two patients with posterior shoulder pain, weakness of inverted glenohumeral external rotation, and isolated infraspinatus atrophy. Magnetic resonance imaging confirmed spinoglenoid notch cysts communicating with type II SLAP lesions in both cases. Both patients underwent arthroscopic labral repair through standard SLAP portals, with intra-articular decompression of the cysts through the pre-existing capsular defect, avoiding additional dissection within the spinoglenoid notch. Postoperative rehabilitation and follow-up imaging demonstrated complete resolution of the cysts, restoration of infraspinatus muscle bulk, and recovery of external rotation strength.

**Conclusions:**

These cases demonstrate that arthroscopic labral repair, combined with indirect cyst decompression, can achieve complete cyst resolution and muscle recovery for selected patients. The findings support the potential reversibility of compression-related muscle atrophy following timely nerve decompression and emphasize the importance of accurate diagnosis and individualized management.

## Introduction

Spinoglenoid notch cysts are an uncommon paralabral lesion frequently associated with superior labrum anterior-to-posterior (SLAP) tears (1). These cysts typically form through a one-way valve mechanism, where synovial fluid extravasates through the labral tear and accumulates in the spinoglenoid notch (2). As the cyst enlarges, it may compress the infraspinatus branch of the suprascapular nerve, resulting in posterior shoulder pain, external rotation weakness, and infraspinatus atrophy. Because of non-specific symptoms, these cysts can be misdiagnosed as common shoulder disorders such as rotator cuff pathology or subacromial impingement (3). Magnetic resonance imaging (MRI) is crucial for confirming the diagnosis and assessing muscle denervation (4, 5).

Various surgical strategies have been reported for spinoglenoid notch cysts associated with SLAP lesions, including open excision, isolated labral repair, and labral repair combined with cyst decompression. Compared with open surgery, arthroscopic techniques are less invasive and allow for simultaneous treatment of the labral pathology (6). However, the optimal approach remains unclear. Specifically, it is essential to achieve sufficient cyst decompression while minimizing manipulation around the suprascapular nerve and reducing recurrence risk (7). In this study, we report two cases of spinoglenoid notch cysts associated with type II SLAP lesions treated with arthroscopic labral repair, combined with indirect cyst decompression through the capsular defect, highlighting a minimally invasive strategy that balances decompression, nerve protection, and recurrence prevention.

## Case presentation

### Case 1

#### Patient information and clinical presentation

A 44-year-old male presented with a 3-year history of right shoulder pain and restricted mobility, without discernible precipitating factors. The pain was described as intermittent, dull, and aching, with no nocturnal exacerbation and no associated limb numbness or morning stiffness. Over the 3 weeks prior to admission, the symptoms had intensified, significantly impairing daily functioning and occupational activities.

#### Physical examination

A physical examination revealed no gross deformity of the right shoulder; however, marked atrophy and depression of the infraspinatus muscle were observed (Figure 1A). Shoulder range of motion was largely preserved (Figures 1B–E), although right-sided external rotation strength was diminished. The O'Brien and O'Driscoll tests yielded positive results, while Speed's and Yergason's tests were negative. Cutaneous sensation of the digits and peripheral circulation were unremarkable. The visual analog scale (VAS) score was 3.

**Figure 1:**
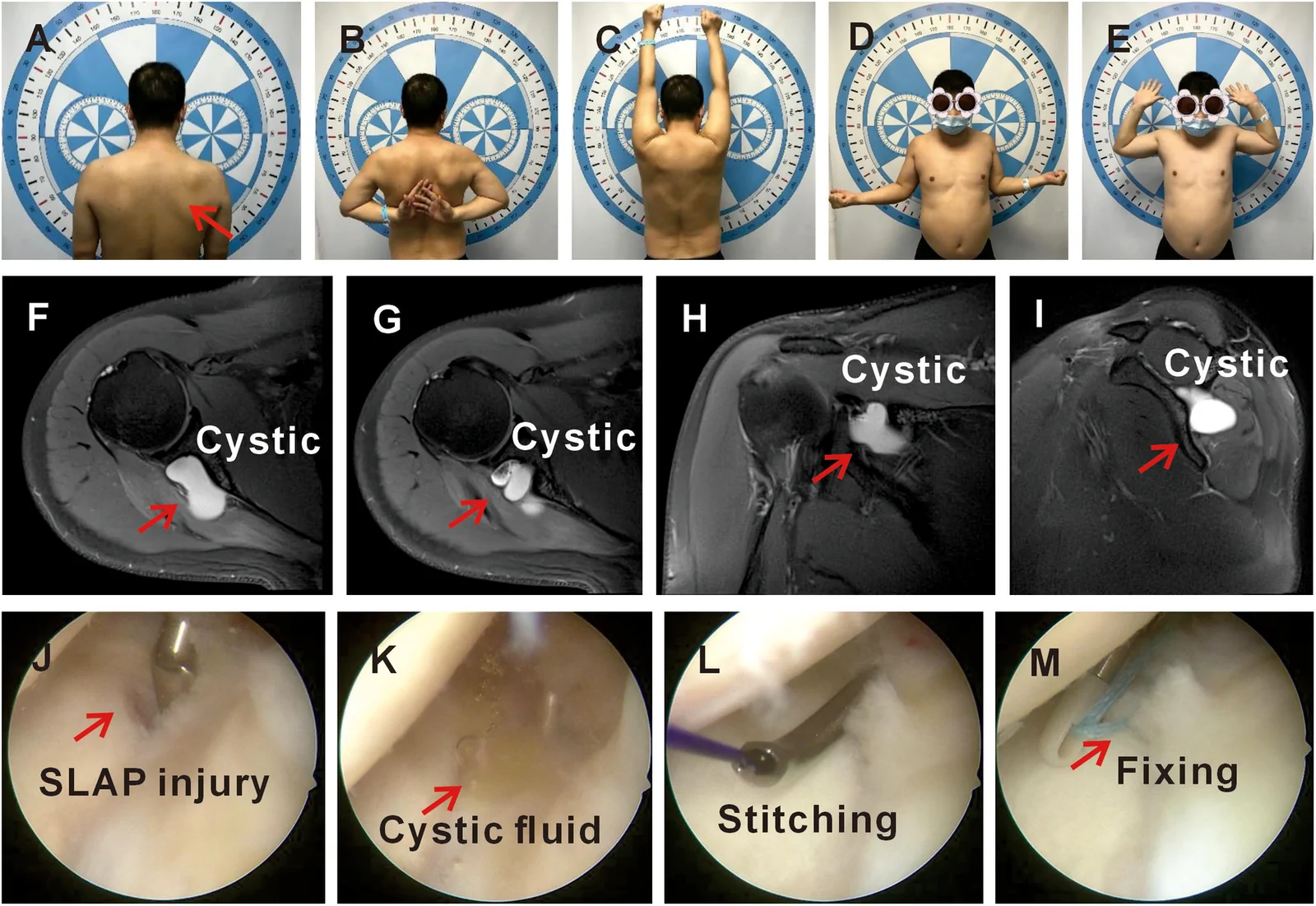
A radiographic and clinical sequence of Case 1. **(A–E)** Preoperative clinical assessment. **(A)** A posterior view showing infraspinatus muscle atrophy and hollowing of the infraspinous fossa. **(B–E)** Shoulder range of motion (ROM) documenting: **(B)** internal rotation to the T12 spinous process, **(C)** forward elevation, **(D)** external rotation at 90° abduction, and **(E)** external rotation at the side. **(F–I)** A preoperative MRI of the shoulder. **(F,G)** Axial, **(H)** coronal, and **(I)** sagittal T2-weighted images revealing a hyperintense ganglion cyst within the spinoglenoid notch, associated with isolated infraspinatus muscle atrophy without fatty infiltration. **(J–M)** Intraoperative arthroscopic findings. **(J)** Debridement of a type II superior labrum anterior–posterior (SLAP) lesion at the superior glenoid rim. **(K)** Cyst decompression yielding thick, yellow gelatinous fluid. **(L)** Suture anchor insertion into the superior glenoid. **(M)** Completed labral repair showing stable fixation and anatomical restoration of the superior labrum.

#### Imaging assessment and preoperative diagnosis

A preoperative MRI demonstrated a well-defined, T2-hyperintense spinoglenoid notch cyst measuring approximately 2.5 cm in maximum diameter. Notably, this lesion was associated with concurrent infraspinatus muscle atrophy, without evidence of fatty infiltration (Figures 1F–I). Correlating these imaging characteristics with the clinical and physical examinations, a preoperative diagnosis of a spinoglenoid notch cyst concomitant with a type II SLAP lesion was established.

#### Surgical management

Surgical intervention was performed under general anesthesia with the patient positioned in lateral decubitus and shoulder traction applied. Intraoperatively, a probe examination confirmed a type II SLAP lesion (Figure 1J). Dissection broadened the labral tear, and a limited debridement of the adjacent cartilage was performed to facilitate healing. Using an anterosuperolateral portal for visualization and a posterolateral working portal, a yellow, gelatinous substance consistent with cyst content was released into the joint as blunt dissection was performed; a concurrent external compression of the infraspinatus fossa expressed intracystic fluid, effecting decompression of the spinoglenoid notch cyst (Figure 1K). The labral tear was subsequently repaired using suture anchor fixation (Figures 1L,M).

#### Postoperative rehabilitation

Postoperatively, the right arm was immobilized in a sling for 4 weeks. Active movement of the elbow and wrist commenced on postoperative day 1, with 0–90° passive shoulder flexion and abduction allowed. Stitches were removed at 2 weeks, at which point external rotation exercises began. The sling was discontinued at 4 weeks, with active shoulder motion initiated thereafter. Gentle muscle strengthening commenced at 6 weeks, advancing to functional muscle strengthening at 12 weeks, with return to sport and MRI re-evaluation scheduled at 6 months.

#### Follow-up and outcomes

On review at 4 weeks, the VAS score had improved to 1. Mild residual atrophy and slight depression of the infraspinatus fossa persisted. Posterior extension, forward flexion, and abduction with external rotation to 90° were mildly restricted, although side-arm external rotation remained intact; external rotation strength was still slightly reduced (Figures 2A–D). At 6 weeks postoperatively, an MRI demonstrated near-complete resolution of the cyst and persistent infraspinatus atrophy without fatty infiltration. At 6 months, a follow-up MRI revealed complete resolution of the cyst, restoration of infraspinatus muscle mass without atrophy or fatty infiltration, and normal examination finding of the infraspinatus fossa with full recovery of muscle strength (Figures 2E–G); the VAS score had decreased to 0 (Table 1).

**Figure 2:**
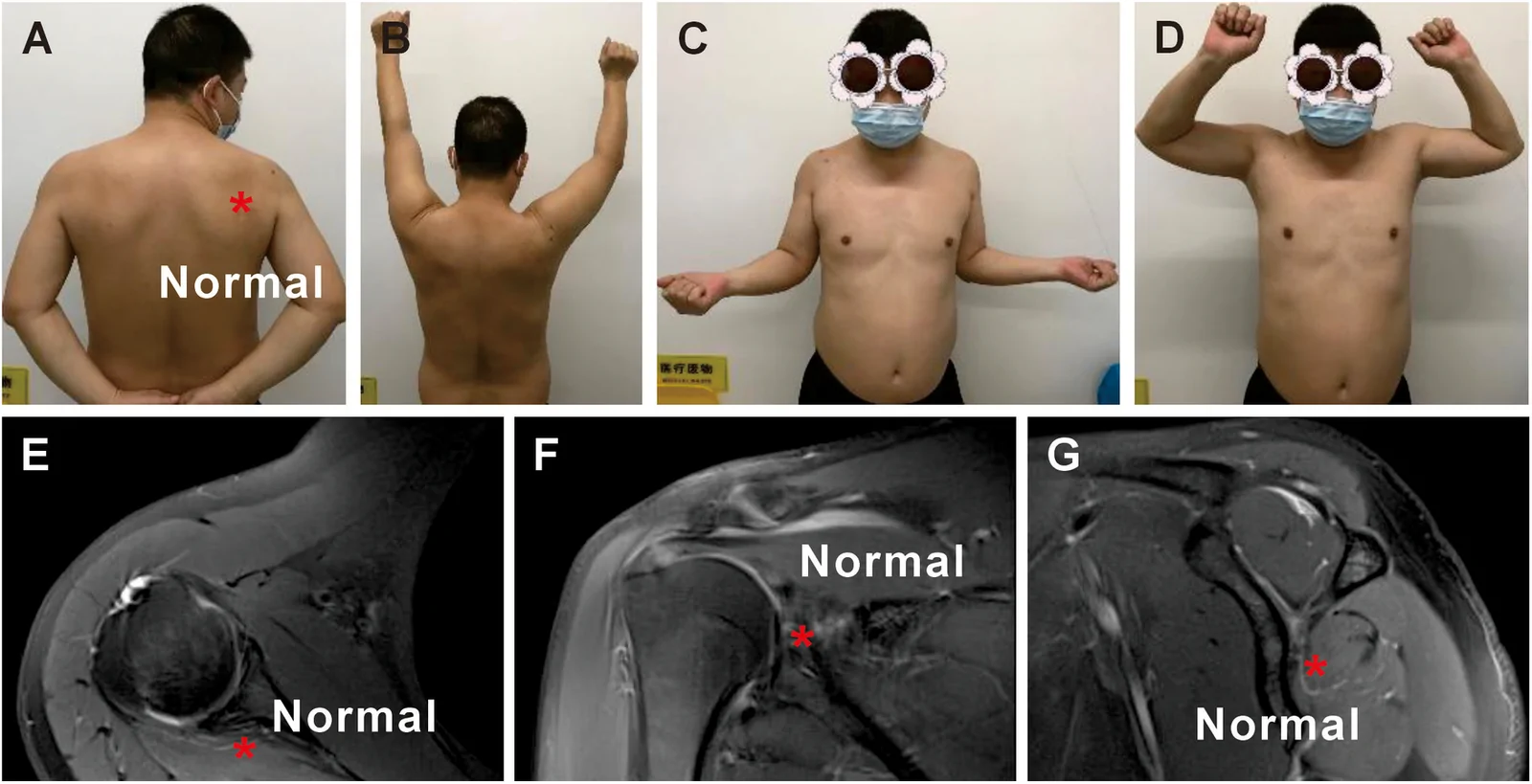
A postoperative clinical and radiographic follow-up for Case 1. **(A–D)** A clinical assessment at 4 weeks postoperatively, demonstrating recovered shoulder range of motion (ROM) with residual limits compared with the contralateral side: **(A)** internal rotation, **(B)** forward elevation, **(C)** external rotation at the side, and **(D)** external rotation at 90° abduction. Persistent mild infraspinatus muscle atrophy and minor hollowing of the infraspinous fossa remain visible. **(E–G)** An MRI at 6 months postoperatively. **(E)** Axial, **(F)** coronal, and **(G)** sagittal T2-weighted views show complete resolution of the spinoglenoid notch cyst, accompanied by partial volume recovery of the infraspinatus muscle belly without fatty infiltration.

**Table 1:** Clinical timeline of presentation, diagnosis, intervention, and follow-up in the two cases.

Time point	Case 1	Case 2
Initial symptom onset	Right shoulder pain developed without obvious cause, persisting for 3 years	Right shoulder pain developed after a twisting injury during golf, persisting for 6 months
Symptom aggravation before admission	Symptoms worsened during the 3 weeks before admission, with limitation in daily activities	Symptoms progressively worsened during the 4 weeks before admission, with functional limitation
Initial clinical findings	Marked infraspinatus atrophy, visible hollow in the infraspinous fossa, reduced external rotation strength, and positive O'Brien and O'Driscoll test results	Mild infraspinatus atrophy, slight depression of the infraspinous fossa, reduced external rotation strength, and positive O'Brien and O'Driscoll test results
Preoperative imaging	An MRI showed a spinoglenoid notch cyst with infraspinatus atrophy and no fatty infiltration	An MRI showed a spinoglenoid notch cyst with infraspinatus atrophy and no fatty infiltration
Preoperative diagnosis	A spinoglenoid notch cyst associated with a type II SLAP lesion and suprascapular nerve compression	A spinoglenoid notch cyst associated with a type II SLAP lesion and suprascapular nerve compression
Surgical intervention	Arthroscopic translabral cyst decompression and SLAP repair with suture anchors	Arthroscopic translabral cyst decompression and SLAP repair with suture anchors
Postoperative rehabilitation	Sling immobilization for 4 weeks; passive shoulder motion initiated on postoperative day 1; external rotation training at 2 weeks; active motion at 4 weeks; strengthening at 6 weeks	Same rehabilitation protocol as Case 1
Early follow-up	At 4 weeks, the VAS pain improved from 3 to 1; mild residual atrophy and weakness persisted	At 4 weeks, the VAS pain score improved from 4 to 1; mild residual atrophy and weakness persisted
Intermediate imaging follow-up	An MRI at 6 weeks showed near-complete cyst resolution, persistent infraspinatus atrophy, and no fatty infiltration	Not available
Final follow-up	At 6 months, an MRI showed complete cyst resolution and restoration of infraspinatus morphology; full recovery of strength; VAS 0	At 6 months, an MRI showed complete cyst resolution and restoration of infraspinatus morphology; full recovery of strength; VAS 0

### Case 2

#### Patient information and clinical presentation

A 39-year-old male presented with a chief complaint of right shoulder pain persisting for 6 months following a golf-related torsional injury. The pain was described as intermittent, dull, and aching, without nocturnal awakening, sensory disturbances, or morning stiffness. Notably, symptom severity had increased over the preceding 4 weeks, exerting a substantial impact on daily life and occupational function.

#### Physical examination

A physical examination demonstrated no overt deformity of the right shoulder but revealed mild atrophy and depression of the infraspinatus muscle, with preserved range of motion. Muscle strength testing indicated diminished external rotation strength of the right shoulder. Positive results of O'Brien and O'Driscoll tests were observed, whereas the results of Speed and Yergason tests were negative. Sensory function and distal perfusion of the digits remained intact. The VAS pain score was 4.

#### Imaging assessment and preoperative diagnosis

A preoperative MRI demonstrated a well-defined, T2-hyperintense spinoglenoid notch cyst measuring approximately 4.0 cm in maximum diameter. Consistent with the first case, infraspinatus muscle atrophy without fatty infiltration was observed (Figures 3A–C). Correlating these imaging features with the clinical presentation, the patient was diagnosed with a spinoglenoid notch cyst concomitant with a type II SLAP lesion.

**Figure 3:**
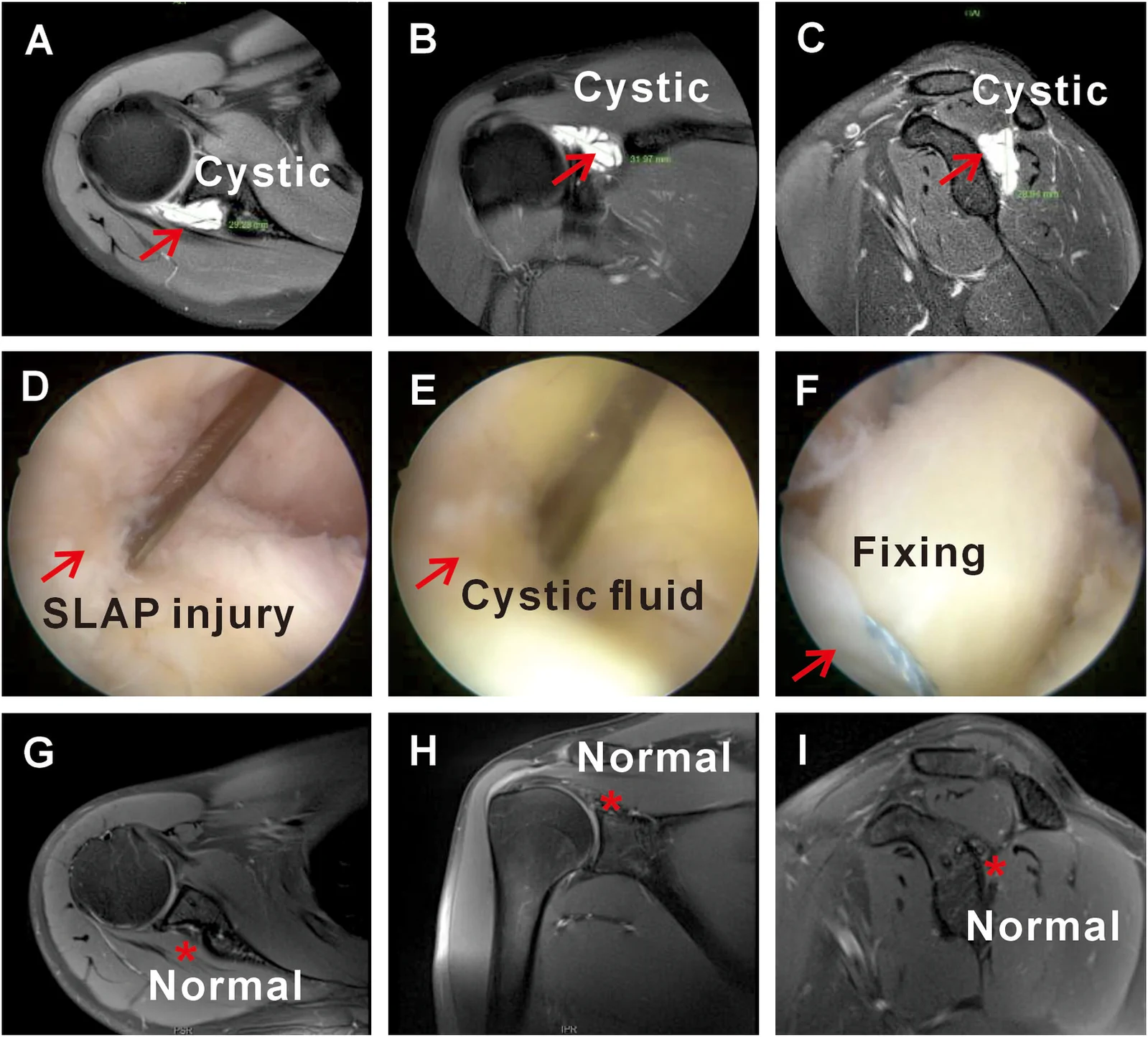
A perioperative imaging and arthroscopic sequence of Case 2. **(A–C)** A preoperative MRI showing a spinoglenoid notch cyst with an associated superior labrum anterior–posterior (SLAP) lesion: **(A)** axial, **(B)** coronal, and **(C)** sagittal T2-weighted views. **(D–F)** Intraoperative arthroscopic findings. **(D)** Identification of the type II SLAP lesion at the superior glenoid rim. **(E)** Cyst decompression with extrusion of yellow gelatinous fluid into the glenohumeral joint. **(F)** Completed SLAP repair following suture anchor fixation. **(G–I)** A postoperative MRI at 6 months showing: **(G)** axial, **(H)** coronal, and **(I)** sagittal T2-weighted views documenting complete cyst resolution and increased infraspinatus muscle volume with no evidence of fatty infiltration.

#### Surgical management and postoperative rehabilitation

The patient underwent surgical intervention as previously described (Figures 3D–F).

#### Follow-up and outcomes

At 4 weeks postoperatively, the VAS pain score improved to 1. A physical examination revealed a persistent mild infraspinatus atrophy and slight depression of the infraspinatus fossa. There was mild limitation in extension, forward flexion, and abduction to 90 degrees with external rotation, while external rotation at the side was relatively preserved, although muscle strength remained diminished. At 6 months postoperatively, an MRI confirmed complete resolution of the cyst and absence of infraspinatus muscle atrophy or fatty infiltration (Figures 3G–I). An examination revealed restoration of the infraspinatus fossa contour and full recovery of muscle strength. The VAS pain score had decreased to 0 (Table 1).

#### Patient perspective

Before surgery, both patients stated that persistent shoulder pain and weakness had affected their daily activities and caused concern, particularly because of the visible hollowing over the posterior shoulder. During follow-up, both patients reported progressive pain relief and improvement in shoulder function after surgery. At the 6-month follow-up, both patients expressed satisfaction with the treatment outcome and felt that the recovery of strength and shoulder contour had significantly improved their confidence in returning to normal activities.

## Discussion

A spinoglenoid notch cyst associated with a type II superior labrum anterior to the posterior lesion is uncommon but clinically important because it may cause suprascapular nerve compression, leading to infraspinatus atrophy and weakness in external rotation. Although both isolated labral repair and labral repair combined with cyst decompression have shown satisfactory outcomes, the optimal surgical strategy remains debated (8, 9). The central issue is how to provide sufficient decompression, while minimizing surgical morbidity and recurrence risk.

In the present report, both patients were treated with arthroscopic repair of the type II SLAP lesion combined with indirect cyst decompression through the existing capsular communication. A postoperative MRI demonstrated complete cyst resolution, and both patients showed substantial clinical improvement. Unlike previously reported techniques that require creation of an additional portal or extensive dissection for direct cyst decompression (10, 11), our procedure used the working pathway already established during SLAP repair. During blunt dissection at the base of the torn labrum, the cyst was decompressed indirectly through the capsular defect. This technique avoided intentional dissection in the spinoglenoid region and therefore may reduce the risk of iatrogenic injury to the suprascapular nerve (12). The distinctive feature of our approach is that decompression was achieved through the existing repair pathway rather than through additional portal creation or deliberate spinoglenoid dissection. From a technical perspective, this approach is less invasive while still addressing both the structural lesion and the compressive pathology.

An important finding in these cases was the marked recovery of infraspinatus bulk and restoration of external rotation strength after cyst resolution. This observation supports the concept that early relief of nerve compression may allow reversal of denervation-related muscle atrophy, particularly when irreversible fatty degeneration has not yet developed (2, 13). Preoperative MRI is therefore crucial not only for confirming the presence of the cyst and associated SLAP lesion, but also for evaluating the degree of muscle involvement. Assessment of muscle atrophy and fatty infiltration provides useful morphological information regarding the severity and potential reversibility of nerve injury (14, 15). In our patients, a preoperative MRI demonstrated infraspinatus atrophy without obvious fatty infiltration, which may explain the favorable postoperative recovery.

These cases also highlight an important diagnostic message. Patients with dull posterior shoulder pain and weakness in external rotation should be evaluated carefully for possible suprascapular nerve compression, even when pain intensity is relatively mild. Visible or palpable infraspinatus atrophy is an important warning sign; however, not all spinoglenoid notch cysts are symptomatic, and some may resolve spontaneously (16). For this reason, clinical suspicion should be combined with targeted MRI evaluation to avoid delayed diagnosis. Early identification is particularly important in patients with preserved muscle quality, as timely surgical intervention may improve the likelihood of functional recovery.

From a pathophysiological standpoint, the favorable outcome observed in these cases further supports the “one-way valve” mechanism of cyst formation. Synovial fluid may pass through the superior labral tear and accumulate outside the joint, gradually forming a ganglion cyst. If only the cyst is treated and the labral lesion is left unaddressed, the underlying mechanism persists and recurrence may occur (8). In this context, labral repair should be considered the cornerstone of treatment. Indirect cyst decompression performed simultaneously may further accelerate cyst collapse and nerve decompression without the added risk associated with extensive direct exposure. This strategy appears particularly suitable for unilocular cysts with clear communication to the labral tear. In contrast, more complex lesions, such as multiloculated cysts, giant cysts, or lesions with ambiguous communication, may still require direct decompression or a different surgical plan.

Postoperative rehabilitation is another essential component of treatment success. Early protected motion is necessary to preserve shoulder mobility while protecting the repaired labrum, whereas progressive strengthening of the external rotators may facilitate neuromuscular recovery and functional restoration (1). This is especially relevant in physically active patients, in whom muscle performance and return to activity are major concerns.

Several limitations in this study should be acknowledged. First, this report includes only two cases, and the case-report design limits the strength and generalizability of the conclusions. Second, no control group or direct comparison with other established techniques, such as isolated labral repair or arthroscopic direct cyst decompression, was available; therefore, no conclusion can be drawn regarding the superiority of this indirect decompression technique. Third, the follow-up period was relatively short, and long-term outcomes, including recurrence, durability of strength recovery, persistent neuropathic deficits, and late shoulder instability, remain uncertain. In addition, electrophysiological studies and standardized functional outcome scores were not available, limiting a more objective assessment of nerve recovery and shoulder function. Finally, the applicability of this technique may be limited to selected patients with clear cyst-labral communication and without advanced irreversible muscle degeneration. Future studies with larger cohorts, comparative designs, longer follow-up, and complementary electrophysiological assessment are needed to better define the indications and long-term efficacy of this technique.

In conclusion, arthroscopic labral repair, combined with indirect decompression of the spinoglenoid notch cyst through the capsular defect, is a safe and effective minimally invasive strategy for selected patients. This technique not only addresses the primary labral pathology but also effectively relieves nerve compression without the risks associated with extensive dissection. Early intervention can lead to the reversal of compression-related muscle atrophy, particularly in patients without irreversible fatty degeneration, and restore shoulder function.

## Data Availability

The raw data supporting the conclusions of this article will be made available by the authors, without undue reservation.
